# Pharmacological Treatment of Neonatal Opiate Withdrawal: Between the Devil and the Deep Blue Sea

**DOI:** 10.1155/2011/935631

**Published:** 2011-05-23

**Authors:** Anthony Liu, Tracey Björkman, Caroline Stewart, Ralph Nanan

**Affiliations:** ^1^Sydney Medical School-Nepean, The University of Sydney, Penrith, NSW 2751, Australia; ^2^Perinatal Research Centre, UQ Centre for Clinical Research, The University of Queensland, Herston, QLD 4029, Australia

## Abstract

Illicit drug use with opiates in pregnancy is a major global health issue with neonatal withdrawal being a common complication. Morphine is the main pharmacological agent administered for the treatment of neonatal withdrawal. In the past, morphine has been considered by and large inert in terms of its long-term effects on the central nervous system. However, recent animal and clinical studies have demonstrated that opiates exhibit significant effects on the growing brain. This includes direct dose-dependent effects on reduction in brain size and weight, protein, DNA, RNA, and neurotransmitters—possibly as a direct consequence of a number of opiate-mediated systems that influence neural cell differentiation, proliferation, and apoptosis. At this stage, we are stuck between the devil and the deep blue sea. There are no real alternatives to pharmacological treatment with opiates and other drugs for neonatal opiate withdrawal and opiate addiction in pregnant women. However, pending further rigorous studies examining the potential harmful effects of opiate exposure in utero and the perinatal period, prolonged use of these agents in the neonatal period should be used judiciously, with caution, and avoided where possible.

## 1. Introduction

Illicit drug use in pregnancy and the associated adverse effects for both mother and child are important public health issues in most developed countries. Recent Australian data has shown that the prevalence of newborns with neonatal withdrawal has surged more than 30-fold over the past two decades, causing a major strain on the health system [1]. Opiates and to a lesser extent barbiturates are the main pharmacological agents administered for the treatment of neonatal withdrawal. In the past, these agents have been considered by and large inert in terms of their long-term effects on the central nervous system [2–5]. However, a growing body of evidence from animal studies and recent clinical studies in children suggests a more cautious approach towards uncritical use of these drugs for neonatal withdrawal.

## 2. Current Approaches to Opiate Treatment of Neonatal Withdrawal

Undoubtedly, neonatal withdrawal is a potentially fatal condition, which requires early recognition and appropriate pharmacological treatment [6]. It is accepted that neonatal withdrawal requires treatment due to its associated morbidity, increased incidence of seizures [7–11], difficulties with weight gain [12–15], increase in infant mortality and sudden infant death syndrome (SIDS) [11, 16–21], and evidence of infant suffering [22]. With these potentially serious consequences, an exclusively nonpharmacological approach to neonatal abstinence cannot be recommended and widespread use of pharmacological agents for neonatal withdrawal is the recognised and accepted practice [6, 13, 23–29]. 

Monitoring of babies exposed to chronic intrauterine amphetamines, cocaine, or opiates is generally recommended for 5–7 days after birth [30], with more than 50% of newborns developing symptoms that require treatment [31]. Treatment in the first instance may include nonpharmacological measures such as swaddling or breastfeeding. Pharmacological interventions using an opiate most commonly or alternatively a barbiturate are then frequently added [26, 29], with pharmacological management of neonatal withdrawal required in 45–80% of cases [32]. A step-up approach usually follows with the aim to titrate medication to an optimal level at which symptoms are controlled. Once effective levels are reached, a weaning process follows which can last from a few days to several months.

## 3. Duration of Opiate Treatment for Neonatal Withdrawal

Length of pharmacological treatment for weaning depends on several factors, such as the maximum dose required or individual withdrawal patterns. It also varies, quite significantly, with the type of weaning strategy employed. For example, rapid weaning involves dose reduction several times per week depending on tight and frequent assessments for withdrawal symptoms. Rapid weaning is generally performed in hospital and can be quite work intensive for nursing staff, parents and carers. On the other hand, slow weaning has become a more frequent practice, with a weekly or fortnightly dose reduction occurring in the home setting. Slow weaning is often used with the rationale of minimising the disruption to maternal-infant bonding by allowing a more gradual reduction of opiates in the home environment, as well as the advantage of reduced costs.

The downside of slow weaning, however, is the potential for more prolonged exposure to either opiates or barbiturates and increased cumulative doses in early infancy. From our own review of 232 cases, the average length of treatment was 49 days for those patients managed in the home setting versus 23 days for in patient weaning. It is important to note that this increased postnatal pharmacological exposure is in addition to the already prolonged exposure to opiates or other illicit drugs whilst in utero. At this stage, there is no data to guide choice of one strategy over another (i.e., rapid versus slow weaning). Therefore, choice of weaning strategy remains subject to the resources and services available at different centres [25, 33].

As a result, the risks of prolonged and increased total cumulative pharmacological exposure to opiates require some attention, particularly given the growing body of evidence that argues *against *opiates as an inert substance for neurodevelopment.

## 4. Effects of Opiates on the Developing Central Nervous System

Opiates exhibit significant effects on the growing brain. This includes direct dose-dependent effects on reduction in brain size and weight, protein, DNA, RNA, and neurotransmitters—possibly as a direct consequence of a number of opiate-mediated systems that influence neural cell differentiation, proliferation, and apoptosis [32]. The animal evidence also suggests that these adverse effects on central nervous system development translate into abnormalities in later animal neurodevelopment and behaviour [34–39].

More specifically opiates appear to interfere with the GABAergic system. While in the mature brain the primary role of the GABAergic system is inhibition, in the immature brain the GABAergic system is predominantly excitatory, a function required for normal brain development. Chronic opiate exposure in utero and in the perinatal period can interfere with normal GABA system development and influence brain excitability and seizure susceptibility [35, 40]. Excessive excitation of brain cells—excitotoxicity—is a well established mechanism of cell injury and death, resulting from an imbalance between excitatory and inhibitory signals. Furthermore, the switch in GABA function is regulated by certain transporter proteins, which have been implicated in excessive excitation and opiate addiction [41].

Superimposed on an already excitatory and compromised environment, prolonged treatment with medications that enhance inhibition in a mature brain could paradoxically lead to potentially damaging levels of excitation in a developing newborn brain. Some evidence of potential adverse effects due to enhanced GABAergic activation includes recent association with impaired cognitive function at 3 years and in utero exposure to valproic acid—an agent that directly targets the GABA system [42].

## 5. Clinical Evidence for Adverse Effects of Opiates in Early Life

Although animal data strongly suggests adverse neurodevelopmental outcomes due to opiate exposure in early life, clinical data in humans remains insufficient, conflicting, and inconclusive [28, 43–48]. So far there is little clinical evidence to support either harmful or benign long-term effects of opiate exposure in utero or the perinatal period. However, the great challenge here is to differentiate between harms directly due to opiates [32, 46, 47, 49] and indirect effects associated with the medical and social complications that cooccur with illicit drug use [2, 43–45, 50, 51].

Many studies do not control for social variables, such as socioeconomic status, early childhood education, maternal education level, and income, home social and psychological environment or family stressors [49, 52–54]. Given the enormous social challenges that face children raised in a drug-exposed environment, it is not possible to draw any meaningful conclusion about the relationship between opiate exposure and harm without controlling for these variables. Some studies attempted to eliminate the social variables by matching children on social environment—for example, by comparing drug-exposed children raised in foster care to non-drug-exposed children [5, 49, 52–56]. However, this introduces additional confounders of foster parents who may be more educated and provide greater support to help overcome the initial adversity faced by the drug-exposed child [55].

In addition to social variables, there are numerous other potential maternal and neonatal confounders to match or statistically control for in order to make valid comparisons between exposed and nonexposed groups [44, 46, 47, 57–59]. This includes, but is not limited to, confounders such as, maternal age, nutrition status, IQ and psychiatric history, neonatal birth weight, gestational age, perinatal complications and congenital and developmental anomalies, and sources of bias, such as selection bias, for example, selecting children who are already suspected of developmental delay [57]. Unfortunately, the majority of available studies are limited or incomplete in their analysis and control for these potential confounders [5, 33, 49, 53–56, 60–63].

In addition, most studies fail to differentiate between exposure to heroin, methadone, or both heroin and methadone, and no studies analyse for the differential effect of additional opiate exposure in the neonatal period. Much of the research also fails to adequately discuss, analyse, or control for the effects of polydrug use [5, 33, 49, 53–56, 60–64]. Even the largest and most well-designed study—the landmark Maternal Lifestyle Study—failed to adequately account for the differential effects of opiate as opposed to cocaine exposure [65, 66]. Overall, it appears that control for the main drug of interest (i.e., opiate versus other drug exposure) is often lost in the complexity of attempting to control for the myriad of maternal, neonatal, and social variables found in this population.

In summary, the current clinical research on neurodevelopmental outcomes of opiate exposure in utero and/or the perinatal period is best limited to small studies and, at worst, methodologically flawed. This highlights the urgent need for well-controlled, preferably large, studies in this area that examine the relationship between opiate use, maternal, neonatal, and social variables, and neurodevelopmental outcomes. The need is particularly urgent given the increasing body of theoretical and animal research suggesting likely harmful effects of opiate exposure on later neurodevelopment and behaviour.

## 6. Are There Alternatives to Opiate Treatment for Neonatal Withdrawal?

The current guidelines certainly suggest that opiates are the most effective pharmacological agent in reducing the symptoms of neonatal withdrawal [6, 13, 24, 26–28]. In particular, it is acknowledged that opiates compared to other pharmacological agents (e.g., phenobarbitone, diazepam, chlorpromazine) appear to reduce the duration of treatment [67–69], the need for a second agent to reduce withdrawal symptoms [8, 68, 69], and the admission rate to neonatal units [68, 69]. They also may reduce the incidence of seizures [27]. It is generally agreed that opiates are superior to other sedative agents such as chlorpromazine and diazepam in controlling abstinence-associated seizures, preventing treatment failure and the need for a second pharmacological agent [24, 25, 27, 28]. They are also considered to have less adverse effects in the neonate compared to sedatives [6, 24]. If a second agent is required, the current recommendations are for the use of phenobarbitone [28] which, when combined with opiates, may assist in the management of seizures, improve behaviour and interaction [70], and reduce the duration of therapy required to minimise symptoms [71]. It is also recommended that, as much as possible, the pharmacological agent used to assist withdrawal should match the agent of in utero addiction (e.g., opiates for opiate addiction, phenobarbitone for amphetamine addiction, etc.) [6, 13, 25]. 

Another pharmacological agent, which demonstrates some promise and may have the potential to be an alternative to opiates, is clonidine, an *α*_2_-adrenergic receptor agonist. Preliminary trials have suggested that clonidine compared to, or in addition to, opiates markedly reduces treatment failure, withdrawal symptoms, duration of therapy, and/or duration of opiate use and maximum dosage required [72–74]. Given the possible safety issues associated with clonidine, however, the guidelines recommend that further clinical trials are required to establish its efficacy and safety in the long term [6, 28].

## 7. Conclusion

At this stage, we are stuck between the devil and the deep blue sea. On the one hand, methadone maintenance treatment for pregnant women who are illicit drug users has consistently been shown to reduce harmful outcomes for both the mother and newborn [3, 32, 58]. Furthermore, there are no real alternatives to pharmacological treatment with opiates and other drugs for neonatal opiate withdrawal and opiate addiction in pregnant women. On the other hand, there is increasing animal and theoretical evidence to challenge the general assumption that opiates are a benign substance to neurodevelopment and, at this stage, there is inadequate clinical research to guide our prescription of opiates for this already vulnerable and at-risk population.

As a result, pending further rigorous studies examining the potential harmful effects of opiate exposure in utero and the perinatal period, prolonged use of these agents in the neonatal period should be used judiciously, with caution, and avoided where possible.
